# CHIP^−/−^-Mouse Liver: Adiponectin-AMPK-FOXO-Activation Overrides CYP2E1-Elicited JNK1-Activation, Delaying Onset of NASH: Therapeutic Implications

**DOI:** 10.1038/srep29423

**Published:** 2016-07-12

**Authors:** Sung-Mi Kim, James P. Grenert, Cam Patterson, Maria Almira Correia

**Affiliations:** 1 Department of Cellular & Molecular Pharmacology, University of California San Francisco, San Francisco CA 94158-2517, USA.; 2 Department of Pathology, University of California San Francisco, San Francisco CA 94158-2517, USA.; 3 The Liver Center, University of California San Francisco, San Francisco CA 94158-2517, USA.; 4 Department of Medicine, Presbyterian Hospital/Weill-Cornell Medical Center, New York, NY 10065, USA.; 5 Department of Pharmaceutical Chemistry, University of California San Francisco, San Francisco CA 94158-2517, USA.; 6 Department of Bioengineering and Therapeutic Sciences, University of California San Francisco, San Francisco CA 94158-2517, USA.

## Abstract

Genetic ablation of C-terminus of Hsc70-interacting protein (CHIP) E3 ubiquitin-ligase impairs hepatic cytochrome P450 CYP2E1 degradation. Consequent CYP2E1 gain of function accelerates reactive O_2_ species (ROS) production, triggering oxidative/proteotoxic stress associated with sustained activation of c-Jun NH_2_-terminal kinase (JNK)-signaling cascades, pro-inflammatory effectors/cytokines, insulin resistance, progressive hepatocellular ballooning and microvesicular steatosis. Despite this, little evidence of nonalcoholic fatty liver disease (NAFLD)/nonalcoholic steatohepatitis (NASH) was found in CHIP^−/−^-mice over the first 8–9-months of life. We herein document that this lack of tissue injury is largely due to the concurrent up-regulation and/or activation of the adiponectin-5′-AMP-activated protein kinase (AMPK)-forkhead box O (FOXO)-signaling axis stemming from at the least three synergistic features: Up-regulated expression of adipose tissue adiponectin and its hepatic adipoR1/adipoR2 receptors, stabilization of hepatic AMPKα1-isoform, identified herein for the first time as a CHIP-ubiquitination substrate (unlike its AMPKα2-isoform), as well as nuclear stabilization of FOXOs, well-known CHIP-ubiquitination targets. Such beneficial predominance of the adiponectin-AMPK-FOXO-signaling axis over the sustained JNK-elevation and injurious insulin resistance in CHIP^−/−^-livers apparently counteracts/delays rapid progression of the hepatic microvesicular steatosis to the characteristic macrovesicular steatosis observed in clinical NASH and/or rodent NASH-models.

The cytosolic E3 ubiquitin (Ub)-ligase C-terminus of Hsc70-interacting protein (CHIP) along with its cognate E2 Ub-conjugating enzyme H5a (UbcH5a) and its cochaperones Hsc70/Hsp40, participates in the Ub-26S proteasome-dependent endoplasmic reticulum-associated degradation (ERAD) of various proteins including the hepatic drug-metabolizing enzymes, cytochromes P450 (P450s)^1^,^2^,^3^,^4^,^5^. CHIP-knockdown in cultured rat hepatocytes stabilizes functionally active P450s CYPs 3A and 2E1 and their inactive ubiquitinated species^4^ (Fig. S1). Such hepatic stabilization of functionally active P450s upon their ERAD-impairment is clinically relevant to P450-dependent drug metabolism and consequent drug-drug interactions, as human CYP3A4 is responsible for the metabolism of >50% of therapeutic drugs^6^. Similarly, human liver CYP2E1 catalyzes the biotransformation of many clinically relevant drugs (ethanol, acetaminophen), carcinogens, and endogenous ketones and fatty acids (FA)^6^. Its ability to bioactivate xenobiotics into toxic/reactive intermediates and its high propensity for generating reactive O_2_ species (ROS) have implicated CYP2E1 in the pathogenesis of toxic liver damage, alcoholic liver disease, nonalcoholic steatohepatitis (NASH), diabetes, and obesity^7^,^8^,^9^,^10^,^11^. Although CYP2E1 is normally less abundant (≈5–7% of human hepatic P450 content) than CYPs 3A (≈30%)^6^, its abnormally elevated basal content (>7%) either via transcriptional induction or protein stabilization in these conditions is thought to predispose and/or abet pathogenesis of liver injury^7^,^8^,^9^,^10^,^11^. Thus tight regulation of CYP2E1 content is clinically desirable. This regulation involves balanced CYP2E1 protein synthesis and degradation via both ERAD and autophagy^2^,^3^,^12^,^13^. Indeed, CYP2E1-stabilization upon autophagic disruption enhances ROS-mediated oxidative stress and cytotoxicity, reducing E47 HepG2 cell-viability^13^.

Although ERAD is also an important determinant of hepatic CYP2E1 content, its physiological relevance and the extent of its dependence on CHIP/UbcH5a/Hsc70/Hsp40-mediated ubiquitination are unknown. CHIP Ub-ligase is physiologically relevant, as its genetic ablation in mice results in premature aging, shortened lifespan, and various pathologies including widespread oxidative damage due to disrupted protein quality control^14^. The markedly increased hepatic lipid peroxidation in 3-month old CHIP^−/−^-mice relative to that in age-matched wild-type (WT; CHIP^+/+^) controls, suggests early oxidative liver damage that within 12 months not only spreads to additional tissues, but also compromises hepatic proteasomal function^14^,^15^. Our findings that CHIP-knockdown increased functional hepatic P450 content^4^ prompted us to examine whether the age-dependent oxidative damage in CHIP^−/−^-livers was due to P450 stabilization.

CYPs 3A and CYP2E1 undergo futile oxidative cycling that generates H_2_O_2_ and other ROS (O_2_^-^ and HO. radicals)^16^,^17^. Herein using relatively selective P450 functional probes we document that CYP2E1 largely, and CYP3A to a lesser extent, contribute to the age-dependent oxidative damage and proteotoxic stress in CHIP^−/−^-livers. Additionally, we document that such chronic CYP2E1-elicited oxidative stress in CHIP^−/−^-hepatocytes is associated with the sustained activation of stress-activated protein kinase (SAPK)/c-Jun NH_2_-terminal kinase (JNK)-signaling cascades, nuclear factor κB (NF-κB) and inflammatory cytokines and chemokines and the Nod-like receptor P3 (NLRP3)-inflammasome, which may significantly contribute to the age-dependent cellular ballooning and microvesicular steatosis observed in CHIP^−/−^-livers. However, in spite of this concatenation of nonalcoholic fatty liver disease (NAFLD)/NASH-like events, little evidence of NAFLD/NASH, as signaled by its hallmark macrovesicular steatosis, was actually found in CHIP^−/−^-livers over the first 8–9 months of life. The significant activation of the hepatic energy- and ROS-sensor 5′AMP-activated protein kinase (AMPK) coupled with the significantly up-regulated expression of epididymal white adipose tissue (EWAT) adiponectin and its hepatic adipoR1/adipoR2-receptors, observed early in 2-month-old CHIP^−/−^-mice relative to that in age-matched CHIP^+/+^-controls, indeed presaged the concurrent up-regulation and activation of the adiponectin-AMPK-forkhead box O (FOXO)-signaling axis. In this, FOXO/FKHR transcription factors, critical nodes at the intersection of the JNK- and AMPK-signaling networks^18^,^19^,^20^,^21^,^22^,^23^, play a key regulatory role to insure that the hepatoprotective adiponectin-AMPK-FOXO-signaling largely prevails over the liver injury-promoting JNK1-pathway, thereby counteracting/delaying any pathogenic progression into NASH in CHIP^−/−^-livers. Our findings reinforce the growing awareness of the beneficial role of adiponectin-AMPK-FOXO-signaling pathway in the pathogenesis of NAFLD/NASH^24^,^25^, suggesting that its therapeutic targeting could be exploited as a management strategy.

## Results

### Hepatic CYP3A and CYP2E1 functional stabilization upon genetic CHIP-knockout induces oxidative stress

Upon Western-immunoblotting (**IB**) analyses, appreciably higher CYP3A and CYP2E1 protein stabilization was observed in cultured CHIP^−/−^-hepatocyte lysates than in CHIP^+/+^-hepatocyte lysates, irrespective of mouse age (Fig. S2B), thereby verifying that CHIP-knockout stabilized both hepatic CYP3A and CYP2E1 content. This increased P450 content in CHIP^−/−^-hepatocytes was functional, as documented by the CYP3A and CYP2E1 functional probes [7-benzyloxy-4-trifluoromethylcoumarin (BFC) and 7-methoxy-4-trifluoromethylcoumarin (MFC), respectively; Fig. S2C].

**Figure 1:**
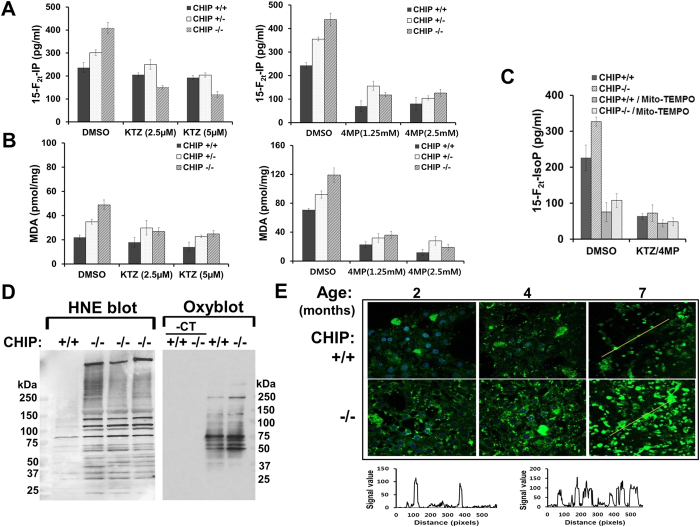
Enhanced oxidative stress stemming from hepatic CYP2E1 and CYP3A stabilization in CHIP^−/−^-livers. Functional contribution of CYP3A (left) and CYP2E1 (right) to hepatic 15-F_2t_-IP production (**A**) and MDA-generation (**B**) in cultured hepatocytes from 2-month-old mice with diagnostic inhibitors KTZ and 4-MP (**C**). Relative contribution of CYP3A and CYP2E1-generated and/or mitochondrial ROS to 15-F_2t_-IP-production assessed with KTZ/4-MP and/or MitoTEMPO as probes. (**D**) IB-analyses of relative HNE-conjugation (left) and oxidized protein-carbonyls (right) in CHIP^+/+^- and CHIP^−/−^-liver lysates. -CT refers to the corresponding controls in the absence of Oxyblot-reagents. (**E**) Confocal immunofluorescence analyses of age-dependent *in situ* HNE-conjugation in hepatocytes cultured from CHIP^+/+^- and CHIP^−/−^-livers. The relative quantification of the HNE-immunofluorescence from 7-month-old CHIP^+/+^- and CHIP^−/−^-hepatocytes^4^ is shown (bottom).

Genetic CHIP ablation significantly increased hepatic 15-F_2t_-isoprostane (15-F_2t_-IP) and malondialdehyde (MDA) basal levels (Fig. 1A–C). The CYP3A inhibitor ketoconazole (KTZ) and CYP2E1 inhibitor 4-methylpyrazole (4-MP) effectively blocked these increases (Fig. 1A,B). When CHIP^+/+^- and CHIP^−/−^-hepatocytes were concomitantly pretreated with both dexamethasone (DEX) and isoniazid (INH) to restore basal CYP3A and CYP2E1 content and then cotreated with KTZ (5 μM) plus 4-MP (2.5 mM), basal 15-F_2t_-IP levels were decreased by 56% and 80%, respectively (Fig. 1C). On the other hand, MitoTEMPO [(2-(2,2,6,6-tetramethylpiperidin-1-oxyl-4-ylamino)-2-oxoethyl) triphenylphosphonium chloride.monohydrate, 100 μM; a mitochondria-targeted antioxidant probe with superoxide/alkyl radical-scavenging properties]^26^ attenuated the elevated 15-F_2t_-IP by 60% in both CHIP^+/+^- and CHIP^−/−^-hepatocytes. MitoTEMPO together with 4-MP and KTZ only slightly enhanced this inhibition, thereby revealing the major functional contribution of CYP3A and CYP2E1 to this 15-F_2t_-IP increase.

Appreciable elevation of 4-hydroxynonenal (HNE)-protein conjugation and oxidized protein side-chain carbonyls provided additional evidence for the relatively enhanced oxidative stress in cultured CHIP^−/−^-hepatocytes (Fig. 1D). *In situ* confocal immunofluorescence of CHIP^−/−^- and CHIP^+/+^-hepatocytes (Fig. 1E) revealed higher HNE levels in CHIP^−/−^-hepatocytes that further increased at 4–7 months (Fig. 1E). Together these findings evince that genetic CHIP-ablation functionally stabilizes hepatic CYP2E1 and CYP3A with consequently heightened intracellular oxidative stress that was largely mitigated by P450 functional inhibitors.

### Age-related pathological changes in CHIP^−/−^- and CHIP^+/+^-hepatocytes: Histological analyses

**Figure 2:**
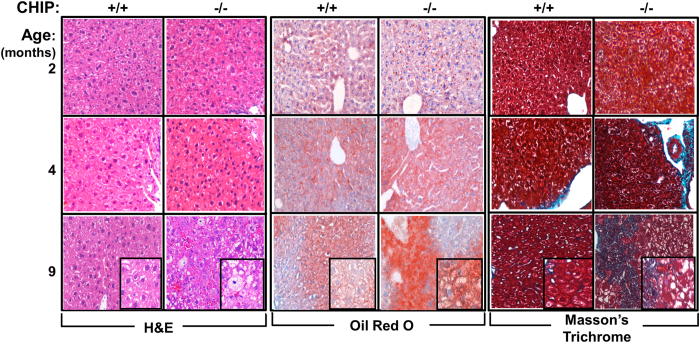
Age-related histological alterations in CHIP^+/+^- and CHIP^−/−^-livers. Representative liver sections stained with H&E (left), Oil red O (middle) and Masson’s trichrome (right) at a 10 μ-magnification view are illustrated. Insets correspond to their respective 20 μ-magnification images.

We characterized the age-related morphological changes in 2-, 4- and 9-month-old CHIP^−/−^- and CHIP^+/+^-mouse livers histologically following staining with hematoxylin and eosin (H&E), Oil red O for detection of lipid accumulation, and Masson’s trichrome for detection of fibrosis (Fig. 2). No great differences in H&E-based histology were detectable at 2- or 4 months between CHIP^−/−^-livers and corresponding age-matched CHIP^+/+^-controls. However with age, hepatocyte ballooning with pyknotic nuclei (characteristic of dying and/or apoptotic cells) was quite marked in 9-month-old CHIP^−/−^-mice relative to age-matched CHIP^+/+^-controls (Fig. 2). No evidence of significant inflammation as demonstrated by lymphocytes and neutrophils in portal or lobular areas, characteristic of clinical NASH, could be found. On the other hand, Oil red O-stained sections revealed “microvesicular” steatosis, but not the macrovesicular steatosis characteristic of clinical NASH and rodent NASH-models^7^,^11^,^27^,^28^, that progressed from 4 to 9 months in CHIP^−/−^-mice relative to age-matched CHIP^+/+^-controls (Fig. 2). Trichrome-stained sections from 9-month-old CHIP^−/−^-livers relative to age-matched CHIP^+/+^-controls revealed central fibrosis, although the sinusoidal pattern usually associated with clinical NASH was not observed. Rather on examining all liver sections, the examining clinical hepatopathologist (JPG) found striking evidence of “*fibrosis due to mild cardiovascular congestion in the central vein stemming from the onset of heart failure*”, a plausible cause of death in these prematurely aging CHIP^−/−^-mice manifesting cardiac hypertrophy and compromised cardiac function^15^. Thus, although these analyses documented age-dependent hepatic lipid accumulation, the characteristics of injury at this stage were clearly different from those of clinical NASH.

### Sustained CYP2E1-mediated oxidative stress in CHIP^−/−^-hepatocytes is associated with the activation of intracellular signaling cascades

To identify any plausible signaling cascades affected by CYP2E1-mediated persistent oxidative stress in CHIP^−/−^-livers, we screened cultured hepatocytes from 2-, 4- and 9-month-old mice using the PathScan Intracellular signaling array kit. This slide-based antibody array provides a broad snapshot of age-dependent activation of signaling modules and/or proapoptotic processes (Fig. S3A). Several key signaling transducers (Fig. S3) activated early (≈2 months) and in a CYP2E1-dependent manner in CHIP^−/−^-livers were thus identified: (i) Mitogen-activated protein kinase (MAPK) JNK, activated by pro-inflammatory cytokines and cellular stresses^8^,^11^,^29^,^30^,^31^,^32^,^33^,^34^, (ii) energy/metabolic sensor AMPK-α1-subunit (AMPKα), activated via Thr172-phosphorylation by elevated intracellular AMP and/or ROS levels^35^, and (iii) insulin/insulin receptor substrate (IRS-1/IRS-2)/phosphatidylinositol 3-kinase-dependent activation of the serine-threonine kinase Akt by 3-phosphoinositide-dependent protein kinase-1 (PDK1) via Thr308-phosphorylation^36^. Insulin-signaling-dependent Akt-activation via Ser473-phosphorylation, on the other hand, was only transiently increased at 4 months, but reverted to basal levels at 9 months in CHIP^−/−^-hepatocytes (Fig. S3). By contrast to JNK, p38MAPK, another similarly activated kinase and the extracellular-signal-regulated kinase (ERK1/2) were markedly activated in CHIP^−/−^-hepatocytes but only at 9 months (Fig. S3B).

Concurrent apoptosis in 9-month-old CHIP^−/−^-hepatocytes relative to age-matched CHIP^+/+^-controls was documented by the activation of the pro-apoptotic signal transducer and activator of transcription 1 (STAT1)-signaling and marked activation of caspase 3 (critical executor of apoptosis) via endoproteolytic cleavage at Asp214 and subsequent cleavage of its principal target polyADP-ribose polymerase 1 (PARP1), involved in DNA repair (Fig. S3). Such enhanced caspase 3-activation was not 4-MP-sensitive and thus CYP2E1-independent. Most likely, consistent with the pyknotic nuclei in 9-month-old CHIP^−/−^-livers (Fig. 2; H&E staining), such apoptosis is inherent to the CHIP-*null* phenotype^14^,^15^.

### Sustained JNK-activation and steatosis in CHIP^−/−^-hepatocytes

**Figure 3:**
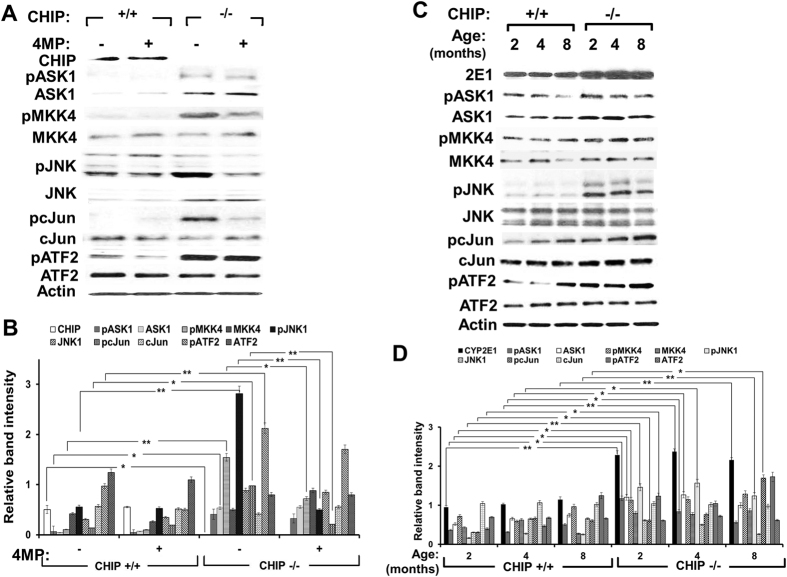
Dependence of JNK-activation pathway transducers on CYP2E1 function. IB-analyses (**A**) and corresponding densitometric immunoquantification (**B**) of the parent proteins and their activated/phosphorylated species in hepatocyte lysates from 2-month-old CHIP^+/+^- and CHIP^−/−^-mice (N = 3 individual mice), treated with or without 4-MP (5 mM) for 2 h before cell-harvest. IB-analyses (**C**) and corresponding densitometric immunoquantification (**D**) of these parameters (N = 3 individual mice) illustrating age-alterations, if any.

Given the marked age- and CYP2E1-dependent JNK-activation in CHIP^−/−^-hepatocytes, we assessed upstream and downstream transducers in the apoptosis signal-regulating kinase (ASK1)-JNK-MAPK-protooncogene c-Jun/activator protein 1 (AP1)-signaling cascade in their native and activated/phosphorylated forms^8^,^10^,^11^,^29^,^30^,^31^,^32^ (Fig. 3A,B). The levels of ASK1, the first transducer in this cascade and its activated form (pASK1) were increased significantly in CHIP^−/−^-hepatocytes at 2 months over age-matched WT-controls. Neither increase was CYP2E1-dependent. MAPK-kinase MKK4 levels were comparable in CHIP^−/−^-hepatocytes and WT-controls; but activated MKK4 (pMKK4) levels were dramatically increased in CHIP^−/−^-hepatocytes relative to WT-controls, in a 4-MP-sensitive manner (Fig. 3). As previously (Fig. S3), the levels of JNK1 (46 kDa) and its activated species (pJNK1) were also markedly increased in CHIP^−/−^-hepatocytes relative to WT-controls, and this JNK-activation was decisively 4-MP sensitive (Fig. 3). The levels of the pJNK-target c-Jun were slightly decreased, whereas those of activated c-Jun (pc-Jun) were significantly increased in CHIP^−/−^-hepatocytes relative to WT-controls. Furthermore, this activation was also 4-MP-sensitive (Fig. 3). On the other hand, the levels of the activating transcription factor 2 (ATF2), another pJNK-target^37^,^38^,^39^, were slightly decreased if at all, but its activation (pATF2) was significantly increased in CHIP^−/−^-hepatocytes relative to WT-controls (Fig. 3). Together these findings reveal that the ASK1-MKK4-JNK-c-Jun- and ASK1-MKK4-JNK-ATF2-signaling cascades were significantly activated upon CHIP-knockout, but only the activation of MKK4, JNK and c-Jun was apparently CYP2E1-dependent. This hepatic pJNK-activation with progressive pc-Jun- and pATF2-activation persisted over 8–9 months (Fig. 3C,D), consistent with the sustained CYP2E1-elicited oxidative stress.

### Up-regulation of hepatic lipogenic genes and pro-inflammatory/inflammatory cytokines/chemokines

The concomitant age-dependent microvesicular steatosis and hepatocyte ballooning observed in CHIP^−/−^-livers relative to corresponding WT-controls (Fig. 2) led us to determine through quantitative real-time polymerase chain reaction (qRT-PCR) analyses of hepatic mRNA expression, whether lipogenic and pro-inflammatory/inflammatory cytokine/chemokine genes were upregulated and/or antilipogenic genes down-regulated (Fig. S4). Indeed, in spite of the rather weak concurrent AktS473-activation (Fig. S3), the expression of both hepatic antilipogenic “insulin-induced genes” *insig-1* and *insig-*2^40^ was significantly down-regulated at 9 months in CHIP^−/−^-livers relative to WT-controls, thereby enhancing hepatic sterol regulatory element-binding protein (SREBP)-proteolytic processing and consequent transcriptional activation of SREBP target genes^40^. Additionally, hepatic *srebp-1 c* and *srebp-2a* gene expression was also concurrently upregulated, thus synergistically upregulating lipogenic target genes responsible for FA-synthesis (*fas1*, *scd-1, acc1*) and lipid-uptake (ATP-binding cassette transporter *abc-a1*), consistent with the observed hepatic microvesicular steatosis^40^.

**Figure 4:**
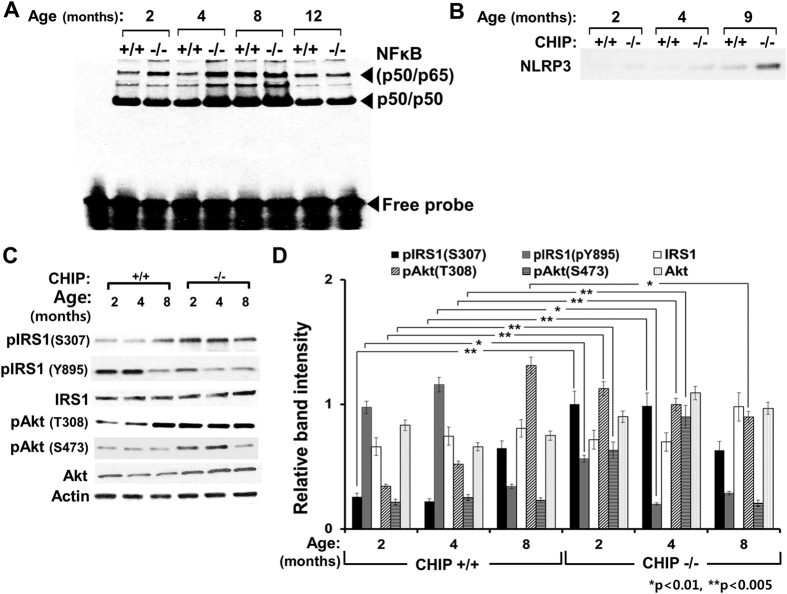
Activation of inflammatory pathways and relative activity of insulin-signaling pathway in CHIP^+/+^- and CHIP^−/−^-livers. NF-κB-activation, monitored through EMSA-analyses of nuclear extracts from 2-, 4-, 8- and 12-month-old intact livers (Experimental Procedures) (**A**). IB-analyses of hepatic NLRP3-component of the NLRP3-inflammasome with mouse age (**B**). (**C**) Age-dependent basal or activated/phosphorylated content of IRS1 and Akt was assessed in cultured hepatocytes via IB-analyses with actin as a loading control. (**D**) The corresponding densitometric immunoquantification relative to actin from 3 individual mouse livers is illustrated.

More importantly, concurrent up-regulation of inflammatory cytokines such as tumor necrosis factor α (TNFα), interleukin 6 (IL-6) and macrophagic chemokine monocyte chemotactic protein 1 (MCP-1) was also significantly detected in CHIP^−/−^-livers as early as 2 months. Their expression along with that of IL-1β remained upregulated at 9 months. In CHIP^−/−^-livers, this TNFα-up-regulation is paralleled by their NF-κB-activation profile^41^. Thus, electromobility shift assays (EMSA) of nuclear extracts from CHIP^−/−^- and CHIP^+/+^-livers revealed the very early relative activation of p65/p50 NF-κB-heterodimers in CHIP^−/−^-livers at 2 and 4 months, that peaked at 8 months but reverted to WT-levels at 12 months (Fig. 4A), possibly due to hepatocyte dropout (*see below*). Similarly, IL-6 up-regulation was also consistent with CYP2E1-dependent pro-inflammatory activation of STAT3-signaling (Fig. S3). Additionally, a decisive elevation of NLRP3 (cryopyrin), an NLRP3-inflammasome component involved in pro-IL-1β-activation to IL-1β^42^ was detected at 9 months in CHIP^−/−^-hepatocytes but not earlier, or in age-matched controls (Fig. 4B). Such up-regulation of inflammatory/pro-inflammatory effectors may stem from the sustained ROS-elicited hepatic JNK-, p38MAPK- and ERK1/2-activation detected in CHIP^−/−^-mice with age. This activation may also account for the relative up-regulation of grp78, an ER-stress marker.

### Relative predisposition to injury of CHIP^−/−^-hepatocytes

Given this remarkable collective pathogenic profile of CHIP^−/−^-hepatocytes, we monitored cytosolic alanine aminotransferase (ALT)-leakage into the medium as a hepatocellular-injury marker over a 24 h-period. Although this was slightly, albeit significantly higher in CHIP^−/−^-hepatocytes from 2-month-old mice relative to those from age-matched WT, little basal cytotoxicity was evident under conditions of routine culture (Williams medium E (WME)/5 days) (Fig. S5). Because all these mice were fed a standard chow-diet, relative predisposition to cell injury conceivably could be elicited upon hepatocyte culture in a reportedly steatogenic methionine-choline deficient (MCD)-WME medium^43^. Although culture in MCD-WME indeed stimulated extracellular ALT-leakage from CHIP^−/−^-hepatocytes, this was only nominally higher than that of similarly cultured CHIP^+/+^-hepatocytes. Comparable findings were observed when cell injury was incited with hepatotoxic acetaminophen concentrations^44^ (Fig. S5). Surprisingly, these findings revealed that in spite of their persistent oxidative stress and activated JNK1-signaling, CHIP^−/−^-hepatocytes were no more predisposed to cell injury than their WT-counterparts.

### Insulin-signaling in the CHIP^−/−^-liver

The PathScan arrays revealed that the relative Akt-Ser473/Thr308-phosphorylation ratio, a plausible index of insulin signaling^45^, actually dropped with age in CHIP^−/−^-livers (Fig. S3). Thus, on the one hand, Akt-activation critical for insulin signaling was apparently impaired in CHIP^−/−^-livers, as inferred from the somewhat feeble activation of glycogen synthase kinase (GSK) 3β, an Akt-target^46^ (*see below*). On the other, the down-regulation of insulin-regulated hepatic *insig-1* and *insig-2* genes signaled adequate insulin-availability in CHIP^−/−^-livers (Fig. S4). These conflicting insulin-dependent responses obfuscated the real status of insulin signaling in CHIP^−/−^-livers and its plausible impact on their relative NAFLD/NASH-susceptibility. Because of this and the significant basal pancreatic CHIP-expression^1^, we directly assessed the functional status of insulin signaling in CHIP^−/−^-livers (Fig. 4C,D). We found that in CHIP^−/−^-hepatocytes although the basal IRS1-expression (Fig. S4) and protein content (Fig. 4C,D) were comparable to those in CHIP^+/+^-controls over the first 8–9 months, IRS1-activation (via Tyr895-phosphorylation) was significantly lower than in age-matched controls at 2 months, and further declined over 4–8 months (Fig. 4C,D). By contrast, IRS1Ser307-phosphorylation was significantly elevated at 2 months in CHIP^−/−^-hepatocytes, but reverted to age-matched WT-levels by 8 months (Fig. 4C,D). However, hepatic insulin signaling as reflected by relative AktSer473/Thr308-phosphorylation, although *per se* not significantly impaired over the first 4 months compared to that in age-matched WT-controls, tended to decline thereafter.

### Activation of hepatic adiponectin-AMPK-FOXO1-signaling axis upon CHIP-ablation

**Figure 5:**
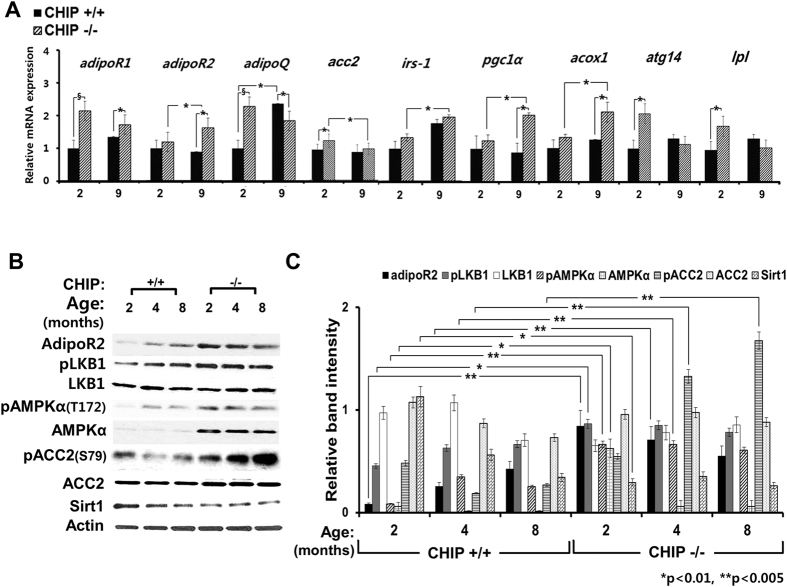
Transcriptional up-regulation of adiponectin-AMPK-FOXO and/or insulin signaling genes and activation of adiponectin-AMPK-FOXO-signaling axis in CHIP^−/−^-livers. (**A**) qRT-PCR analyses of total RNA extracted from intact CHIP^+/+^- and CHIP^−/−^-livers [adiponectin receptors (*adipoR1, adipoR2*), *acc2, irs-1* and FOXO-regulated genes (*pgc1*α*, acox1, atg14, lpl*)] or adipose tissue (*adipoQ*) at 2 or 9 months. Relative mRNA expression was quantified as described (Methods, Table S1). Statistical significance between the values shown at p < 0.001 (*) or p < 0.005 (§). (**B**) Age-dependent expression of various transducers in this pathway detected through IB-analyses and (**C**) corresponding densitometric immunoquantification of individual hepatocytes cultures from N = 3 CHIP^−/−^- and CHIP^+/+^-livers. AMPKα-activation via Thr172-phosphorylation was monitored in cultured CHIP^−/−^- and CHIP^+/+^-hepatocytes.

For reasons discussed above, we examined whether the significant CYP2E1-dependent AMPK activation (pAMPKα1) was associated with concurrent hepatic activation of the adiponectin-AMPK-FOXO-signaling, an event that could potentially delay NAFLD/NASH-onset. Indeed, the expression of hepatic adiponectin receptors (*adipoR1/adipoR2*) and the adipocyte adipokine adiponectin (*adipoQ*) was significantly up-regulated at 2 months in CHIP^−/−^-livers relative to age-matched WT, but the latter began to decline at 9 months (Fig. 5A). Although the content of the AMPK-activating kinase, liver kinase B1 (LKB1) in CHIP^−/−^- and CHIP^+/+^-hepatocytes was comparable, its activated (pLKB1) levels were relatively increased over 2–8 months in CHIP^−/−^-hepatocytes (Fig. 5B,C). Consistent with this LKB1-activation, a significant enhancement of AMPK-activation (pAMPKα) was concurrently observed in CHIP^−/−^-hepatocytes relative to age-matched WT-controls (Fig. 5B,C). This AMPKα-activation in CHIP^−/−^-hepatocytes was also 4-MP-sensitive and thus CYP2E1-dependent (Fig. 5B,C). Predictably, this AMPK-activation was associated with the marked Ser79-phosphorylation of acetyl-CoA carboxylase (ACC2), its diagnostic probe^35^,^47^, thereby attesting to its physiological relevance (Fig. 5B,C).

**Figure 6:**
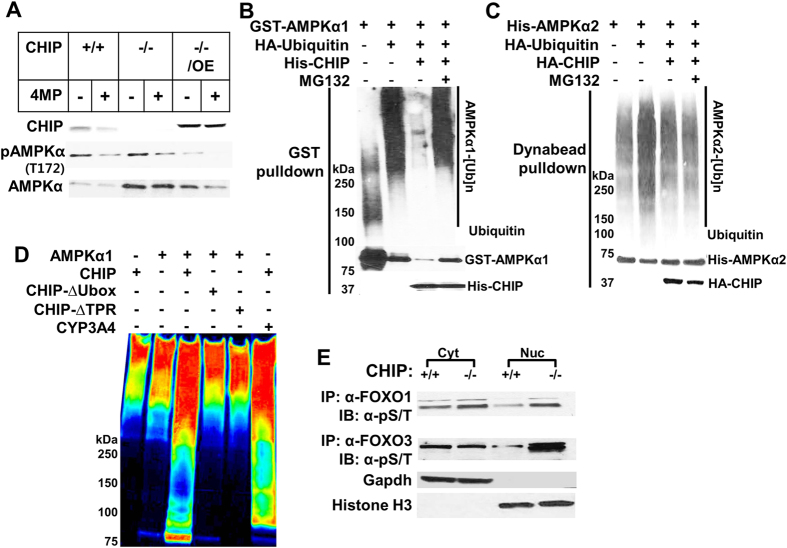
AMPKα1 but not AMPKα2 is a CHIP-substrate. (**A**) The content of AMPKα-subunit and its activated species (pAMPKα) was monitored in the presence or absence of CYP2E1 inhibitor 4MP. In parallel, some CHIP^−/−^-hepatocytes were also transfected with a CHIP-expression plasmid for 36 h, before harvesting. HEK293T cells were cotransfected with GST-AMPKα1- or [His]_6_AMPKα2, HA-Ub- and/or [His]_6_CHIP-expression vectors ± MG132 (10 μM), followed by GSH-Sepharose- (**B**) or Dynabead-His_6_- (**C**) pull-down, and subsequent IB-analyses. *In vitro* AMPKα1- or CYP3A4 (positive control)-ubiquitination (**D**) in a functionally reconstituted CHIP-system with purified CHIP, its U-box- or TPR-deleted mutant, and subsequent IB-analyses with anti-HA antibody and Typhoon visualization^4^. Color wheel intensity code: Red > orange > yellow > green > blue > indigo > violet. (**E**) Hepatic nuclear and cytosolic distribution of activated (pS/pT) FOXO1/FOXO3-species in CHIP^−/−^-livers. Liver lysates were subjected to subfractionation as detailed (Experimental Procedures). Nuclear or cytosolic subfraction was immunoprecipitated (IP) with either FOXO1- or FOXO3-antibody, followed by IB-analyses with an anti-pSer/pThr-antibody. GAPDH and Histone H3 were used respectively as markers of relative cytosolic and nuclear fraction purity.

Remarkably, the basal protein content of AMPKα was distinctly increased in CHIP^−/−^-hepatocytes (Fig. 5), and this increase was attenuated upon exogenous CHIP-overexpression (Fig. 6A). This suggested that hepatic AMPKα is either a CHIP-substrate, or requires CHIP as a chaperone for its degradation. To examine the first possibility, we co-transfected HEK293T cells with glutathione S-transferase (GST)-AMPKα1-, haemagglutinin (HA)-Ub-, and/or [His]_6_CHIP-plasmid vectors, singly or in combination (Fig. 6B). GSH-Sepharose pull-down coupled with IB-analyses revealed that AMPKα1 was indeed intracellularly ubiquitinated when all three vectors were cotransfected (Fig. 6B). By contrast, similar coexpression of HA-CHIP and HA-Ub failed to enhance AMPKα2-ubiquitination (Fig. 6C). Significant AMPKα1-ubiquitination was also detected upon cotransfection of just GST-AMPKα1 and HA-Ub, presumably due to endogenous CHIP and/or other putative E3 Ub-ligases (*see below*). Furthermore, such AMPKα1- but not AMPKα2-ubiquitination could be enhanced upon treatment of the cotransfected cells with the proteasomal inhibitor MG132 (Fig. 6B,C). Incontrovertible evidence was provided by the *in vitro* ubiquitination of AMPKα1-isoform in a functionally reconstituted CHIP-system (Fig. 6D). Such CHIP-mediated AMPKα1-ubiquitination required both the CHIP-cochaperone-interacting tetratricopeptide repeat (TPR) and Ub-ligase U-box-catalytic subdomains (Fig. 6D). To our knowledge, this is the first evidence that in contrast to AMPKα2, hepatic AMPKα1-isoform is a target of both CHIP-ubiquitination and proteasomal degradation.

In mammalian cells, activated AMPK phosphorylates FOXO (FOXO1, FOXO3, FOXO4 and FOXO6)-transcription factors^18^,^23^, well-established CHIP substrates^48^, and also increases their expression and protein stability^18^,^23^,^49^. Indeed, we found that CHIP^−/−^-hepatocytes exhibited not only a relatively higher total cellular phosphorylated FOXO1 and FOXO3 content than CHIP^+/+^-controls, but also greater nuclear retention of their transcriptionally active phosphorylated species as evident upon cell-subfractionation (Fig. 6E). Such enhanced nuclear retention/segregation apparently protects FOXOs from proteasomal degradation, and this protein stability of the AMPK-preferred target FOXO3 was particularly striking (Fig. 6E). As expected^50^, this enhanced FOXO-stability was associated with the transcriptional up-regulation of peroxisome proliferator-activated receptor-γ-coactivator *pgc1*α, and its target acetyl-CoA oxidase (*acox1*) (Fig. 5A), consistent with the transcriptional up-regulation of energy, lipid metabolism and oxidative stress-resistance genes upon AMPK-mediated FOXO-activation^18^,^19^,^20^,^21^,^22^,^23^. Such enhanced hepatic FOXO activation was also associated with the up-regulation of their target *Atg14* and *lpl* autophagic/lipophagic genes^23^,^51^,^52^ in CHIP^−/−^-livers (*see below*) (Fig. 5A).

### Age-dependent progression of “microvesicular” to macrovesicular steatosis in CHIP^−/−^-livers: Onset of NASH?

**Figure 7:**
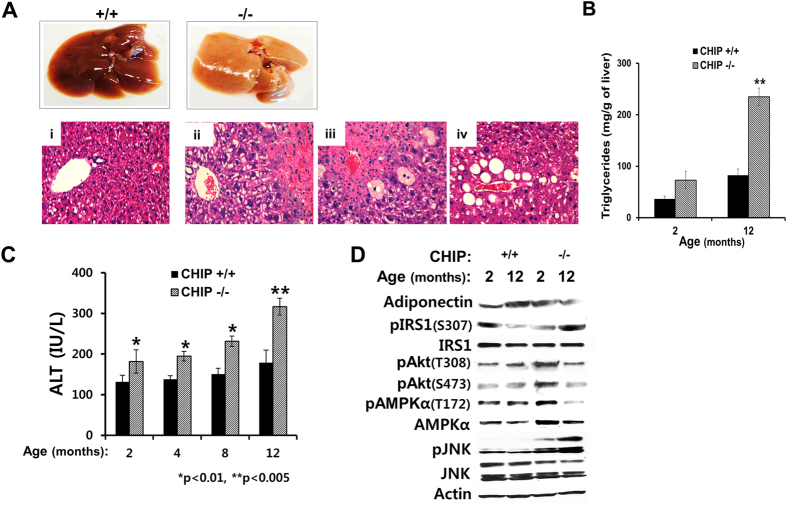
CHIP^−/−^-liver progression to NAFLD/NASH-pathology with age. Freshly excised livers from 12-month-old CHIP^+/+^- and CHIP^−/−^-mice and corresponding H&E staining of sections (**A-i**,**ii**). CHIP^−/−^-liver with central venous congestion (**ii**) and hallmark NASH-hepatocellular ballooning (**iii**) and macrovesicular steatosis (**iv**). (**B**) Relative age-dependent triglyceride content of intact CHIP^−/−^- and CHIP^+/+^-livers. Statistical significance between 12-month-old CHIP^−/−^-livers and corresponding 2-month-old-livers or age-matched WT-controls p < 0.005 (**). (**C**) Relative serum ALT-levels of 12-month-old CHIP^−/−^- and CHIP^+/+^-mice. (**D**) Aging-dependent alterations of various key indices of the adiponectin-AMPK-, JNK- and insulin-signaling pathways in CHIP^−/−^-liver. Except for adiponectin levels monitored in EWAT, all the other parameters were monitored in lysates from CHIP^−/−^- and CHIP^+/+^-livers.

Gross inspection of surviving 12-month-old CHIP^−/−^-mice revealed that rather than the deep red exhibited by age-matched WT-controls, their livers were typically light brown in color (Fig. 7A), indicative of fat accumulation. This was verified by the ≈3-fold higher triglyceride content of 12-month-old CHIP^−/−^-livers, than that of either age-matched WT-controls or 2-month-old CHIP^−/−^-livers (Fig. 7B). Parallel H&E analyses revealed that the microvesicular steatosis observed in 2-month-old CHIP^−/−^-livers (Fig. 2) had now progressed to the central macrovesicular steatosis characteristic of NAFLD/NASH livers (Fig. 7A-iv). The remarkably high prevalence of ballooned cells (Fig. 7A-iii), another cardinal feature of NAFLD/NASH^32^, and central venous-congestion (Fig. 7A-ii,iii), along with the significant rise of serum ALT-levels in these 12-month-old CHIP^−/−^-livers (Fig. 7C), suggested that the protective mechanisms operating at earlier ages were now becoming defunct. Indeed, the elevated levels of adiponectin and pAMPK observed in 2-month-old CHIP^−/−^-livers had appreciably declined at 12 months and were lower than those of age-matched WT-livers (Fig. 7D). By contrast, the activation of JNK1 as well as JNK2 kept progressing beyond that observed in 2-month-old CHIP^−/−^-livers, with a consequent further elevation of IRS1-Ser307-phosphorylation (Fig. 7D). These findings are consistent with an age-dependent disruption of the beneficial adiponectin-AMPK-FOXO- and insulin-signaling pathways that apparently protected younger CHIP^−/−^-livers from NAFLD/NASH.

## Discussion

The cochaperone/E3-ligase CHIP actively participates in newly synthesized and/or misfolded protein-folding and, when that fails, in protein-triage via ERAD^1^,^2^,^3^,^15^. Thus CHIP is vital to cellular proteostasis and quality control. Not surprisingly, its genetic ablation in mice not only results in widespread oxidative/proteotoxic stress in organs including the liver, but also premature aging and shortened lifespan^14^,^15^. We detail herein that such persistent oxidative stress in the CHIP^−/−^-livers is predominantly due to the functional stabilization of CYP2E1 (and CYPs 3A to a lesser extent), which rely on CHIP for their ERAD. Studies with a specific quencher of mitochondrial ROS (MitoTEMPO) coupled with CYP2E1/CYP3A functional inhibitors (4-MP/KTZ) as probes (Fig. 1C) implicate both microsomal and mitochondrially-translocated P450s as the principal ROS-generators in CHIP^−/−^-livers. Persistent generation of injurious ROS rapidly induces oxidative stress that is sustained, triggering the activation of pathogenic signaling cascades, specifically the ASK1-MKK4-JNK1-c-Jun/AP-1-pathway.

Hepatic CYP2E1 elevation with associated oxidative stress and consequently sustained JNK-activation has been implicated in NAFLD/NASH^7^,^8^,^9^,^10^,^11^,^30^,^31^,^32^,^33^,^34^,^53^,^54^. These are also the common pathogenic denominators of high fat (HF)- and MCD-dietary NAFLD/NASH mouse models^26^,^27^,^32^. These models typically exhibit the activation of JNK-c-Jun/AP-1-signaling pathway along with progressively worsening hepatic lipid peroxidation, macrovesicular steatosis, triglyceride accumulation, inflammation, injury and apoptosis that are either prevented or attenuated upon genetic ablation of hepatic JNK1 but not JNK2 gene^55^.

**Figure 8:**
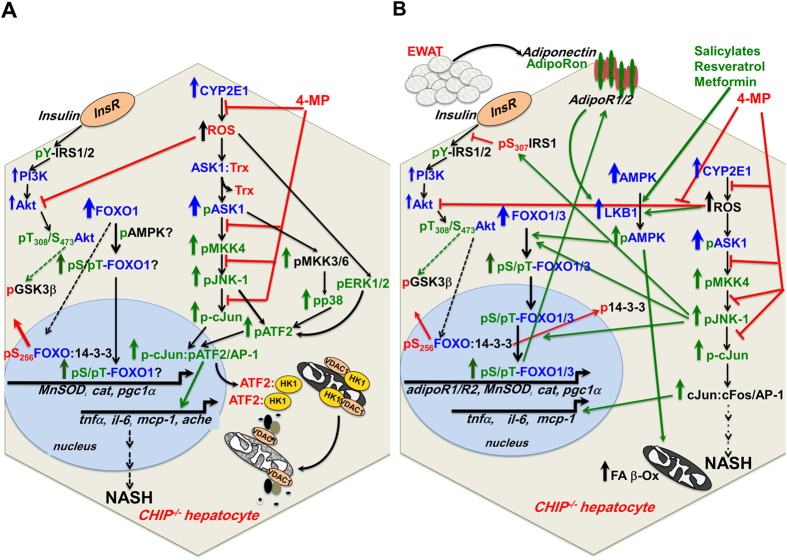
Beneficial adiponectin-AMPK-FOXO-signaling activation overrides the injurious CYP2E1-ROS-JNK-signaling activation in CHIP^−/−^-liver: Therapeutic implications. (**A**) ROS-elicited activation of ASK1-JNK-c-Jun- and ASK1-JNK-ATF2-signaling pathways in CHIP^−/−^-hepatocyte: CYP2E1-dependence. Hepatic proteins known/shown to be stabilized upon CHIP-ablation are shown in blue. Proteins activated via phosphorylation are shown in green. Steps inhibited by 4-MP and thus CYP2E1-dependent are indicated by red stop-lines. Nuclear pc-Jun-pATF2-heterodimerization results in the transcriptional up-regulation of pro-inflammatory factors/cytokines, and specifically in *ache-*expression. On dissociation, pATF2 is dephosphorylated and escapes the nucleus whereupon it interacts with outer mitochondrial membrane hexokinase 1 (HK1), disrupting the HK1-voltage-dependent anion channel 1 (VDAC1) dimer and resulting in leakage of intramitochondrial contents. (**B**) The intersecting hepatic JNK-, insulin-, and adiponectin-AMPK-FOXO signaling pathways that converge on FOXO-transcription factors and proteins stabilized upon CHIP-ablation are depicted, but several relevant players (ATF2, p38, ERK1/2, *ache*, etc.) shown in A are excluded for simplification. Pharmacological agents (AdipoRon, salicylates, metformin, etc) that are known adiponectin-AMPK-FOXO-signaling activators may be beneficial in counteracting/delaying NAFLD/NASH. EWAT, epididymal white adipose tissue; InsR, insulin receptor; Trx, thioredoxin. See Discussion for greater details.

Although CHIP^−/−^-mice reportedly show reduced whole body-fat storage^14^, yet quite early at 2 months of age, their livers exhibited “microvesicular” steatosis, even though they were fed a standard chow-diet rather than a typically conducive steatogenic-diet. Concurrently, CHIP^−/−^-livers documented a significant activation of ASK1-MKK4-JNK1-c-Jun/ATF2-signaling, stemming from (i) increased content of ASK1, the priming member of this signaling cascade and a known CHIP-ubiquitination target^56^, and (ii) oxidative stress-elicited ASK1-activation^57^. Both these events would synergistically amplify the activation of JNK-signaling in CHIP^−/−^-livers (Fig. 8A), leading to the activation of both pc-Jun-AP-1- and pc-Jun-ATF2-signaling pathways, very early on, and in a CYP2E1-dependent manner. It is presently unclear whether the apparent CYP2E1-independence of ASK1 activation is due to its disrupted CHIP-dependent proteasomal degradation that would enable pASK1 to persist beyond the short (2 h) duration of 4-MP-treatment, or whether along with the similarly CYP2E1-independent ATF2 activation, it is an inherent feature of the CHIP^−/−^-phenotypic apoptosis^14^,^15^.

Similar stabilization of the ubiquitously expressed CYP2E1 in nonhepatic CHIP^−/−^-tissues (i.e. adipocytes) would also magnify this JNK1-activation response, thereby transcriptionally up-regulating pro-inflammatory/inflammatory cytokines/chemokines such as TNFα, IL-6, and MCP-1 detected early (2 months), with subsequent IL-1β up-regulation at >9 months. Remarkably, TNFα coupled with elevated ROS can synergistically foster vicious, upward-spiraling pathogenic cycles entailing activation of JNK1-/p38- and IKK-NF-κB-signaling via Complex I as well as proapoptotic caspases 8 and 3 via Complex II^8^,^41^,^53^,^54^. In spite of this potentially beneficial TNFα-elicited antiapoptogenic NF-κB activation, the singular convergence of TNFα-elicited JNK1-/p38-activation, the concurrent ROS-mediated ASK1-activation and ROS-mediated inactivation of phosphatases [i.e. MAPK phosphatase 1 (MKP-1); that normally deactivate pJNK1 and pp38]^8^, would greatly exacerbate and prolong hepatic JNK1-/p38-activation, progressively leading to mitochondrial dysfunction and necrotic/apoptotic hepatocyte death. Additionally, the reciprocal positive feed-forward interdependence between JNK1 and ROS, whereby ROS not only activate JNK1 but are also elevated by it^8^,^54^, would further heighten the inherent oxidative stress. This concerted pathogenic scenario triggered by CYP2E1-stabilization and sustained oxidative stress would account for the age-dependent progressive steatosis, nuclear pyknosis and hepatocyte ballooning observed at 9 months in CHIP^−/−^-livers relative to age-matched controls.

Although JNK, p38MAPK and ERK1/2 all activate ATF2, the marked ATF2-activation in 2-month-old CHIP^−/−^-livers most closely mirrored their temporal JNK1-activation profile. Similarly, the concurrent temporal activation of ASK1-JNK1-ATF2- and ASK1-JNK1-c-Jun-signaling cascades suggests that pATF2 preferentially interacts with the AP-1-component pc-Jun for its nuclear transcriptional activation of pro-inflammatory cytokines/chemokines and apoptotic effectors i.e. acetylcholinesterase (*ache*)^37^ (Fig. S4). With age (2–9 months), oxidative stress and JNK1-activation progress, p38MAPK- and ERK1/2-activation becomes evident, and inflammatory cytokines accumulate in CHIP^−/−^-livers. Such severe sustained stress is expected to eventually lead to ATF2-mediated disruption of the outer mitochondrial membrane permeability^38^,^39^, with leakage of intramitochondrial components (i.e. cytochrome c), promoting cell death (Fig. 8A). Given this serious pathogenic potential, the relative resistance of the CHIP^−/−^-livers to acute cell injury (Fig. S5) is indeed remarkable.

This is all the more remarkable, given that insulin-signaling in CHIP^−/−^-livers relative to age-matched WT-controls was relatively defective as judged by two telltale indices (i) the relatively increased IRS1-S307-phosphorylation secondary to the sustained JNK1-activation^58^,^59^, with correspondingly reduced IRS1-Y895-phosphorylation required for efficient insulin-signaling; and (ii) the declining relative AktSer473/Thr308-phosphorylation after 4 months, duly mirrored by the corresponding phosphorylation profile of its GSK3β-target^45^,^46^ (Fig. S3). By these criteria, CHIP^−/−^-livers became insulin-resistant around 4 months of age. Conditions such as Type 2 diabetes and obesity that promote cellular insulin resistance and contribute to the “metabolic syndrome” are generally known to aggravate the clinical severity of NAFLD/NASH, worsening its prognosis^24^,^30^,^31^,^32^. However, in spite of all these cardinal NAFLD/NASH pathognomonic features of CHIP^−/−^-livers, little evidence exists of their simple hepatic steatosis progressing rapidly into NAFLD/NASH steatohepatitis. Apparently, CHIP^−/−^-mice only succumb to NAFLD/NASH much later in life ≈9–12 months. We posit that the early activation of the antisteatogenic adiponectin-LKB1-AMPK-FOXO-signaling axis effectively protects the CHIP^−/−^-liver from aggravated NAFLD/NASH-manifestations.

Indeed, LKB1, one of the two AMPK-activating kinases, and an established CHIP-substrate^60^,^61^, was consistently activated (pLKB1) in CHIP^−/−^-mice relative to age-matched WT-controls (Fig. 5B). This coupled with the relatively elevated basal AMPKα-content resulted in marked AMPK-activation (pAMPKα) in CHIP^−/−^-livers (Fig. 5B). Herein we document for the first time to our knowledge, that AMPKα1, a major hepatic AMPKα catalytic-subunit-isoform, is indeed a *bona fide* CHIP-substrate (Fig. 6). By contrast, AMPKα2, the predominant cardiac isoform (sharing 75%-sequence identity and 85%-sequence similarity with hepatic AMPKα1-isoform)^61^,^62^,^63^ is not (Fig. 6C). Furthermore, AMPKα2 is stabilized through CHIP-chaperone-function, being functionally lost upon CHIP-ablation^61^,^63^. However, unlike AMPKα2, hepatic AMPKα1-protein is actually stabilized upon CHIP-ablation. Notably, E3-ligases other than CHIP, known to polyubiquitinate AMPKα2-, β1- and β2-isoforms (*reviewed in* ref. 63), may have also contributed to the basal AMPKα1- and AMPKα2-ubiquitination (Fig. 6D).

A significant additional contributor to AMPK-activation in CHIP^−/−^-livers is the concurrently elevated oxidative stress, that would further amplify this activation bimodally through: (i) the canonical AMPK energy-sensing mechanism stemming from ROS-elicited oxidative inactivation of mitochondrial ATP-synthesis and potential ATF2-mediated mitochondrial disruption, with consequently increased cellular AMP/ATP ratios^35^, and (ii) a “*non-canonical*” activation, wherein ROS trigger the oxidation and subsequent glutathionylation of two conserved AMPKα-subunit Cys-residues^21^,^35^. One notable consequence of this magnified AMPK-activation in CHIP^−/−^-livers would be its significant anti-steatogenic action via pSer79ACC2-mediated attenuation of malonyl-CoA production, thereby derepressing carnitine palmitoyltransferase 1 activity, and enhancing mitochondrial FA uptake and β-oxidation^47^.

Another equally relevant consequence is the marked downstream activation of the redox-sensing FOXO-transcription factors^18^,^19^,^20^,^21^,^22^,^23^. AMPK-mediated C-terminal Thr649-phosphorylation of FOXO1 would reduce its affinity for 14-3-3 scaffold proteins, thereby enhancing its nuclear retention and protein stability^20^,^49^, and thus its transcriptional activation of oxidative stress resistance genes (i.e. Mn/Cu-superoxide dismutase, catalase, peroxiredoxins and peroxidases), *pgc-1*α, as well as hepatic cell surface adiponectin receptors (*adipoR1/adipoR2*)^20^,^21^,^23^. Such up-regulated expression of adipoR1/adipoR2 receptors in the CHIP^−/−^-livers coupled with the increased EWAT adiponectin production would enhance hepatic adiponectin-sensitivity, thereby further stimulating the LKB1-AMPK-FOXO-signaling, and establishing a feed-forward mechanism to counteract the inherent oxidative stress through transcriptional activation of oxidative stress resistance genes^19^,^64^,^65^,^66^ (Fig. 8B). Three additional features further amplify this adiponectin-mediated activation of the LKB1-AMPK-FOXO-signaling cascade and its corresponding anti-oxidative stress response in CHIP^−/−^-livers: First, FOXO1, itself being a CHIP substrate^48^, would be stabilized in these livers. Second, activated JNK1 by directly phosphorylating FOXO-proteins (other than FOXO1) at C-terminal Thr-residues, would enhance their transcriptional activity^21^,^67^. Third, activated JNK1 would concurrently also promote the phosphorylation and subsequent proteasomal degradation of 14-3-3 scaffold proteins that mediate the nuclear export of FOXO-proteins into the cytosol in response to insulin-mediated Akt-activation^20^,^36^. These synergistic features would greatly magnify the nuclear retention/segregation of FOXO-proteins, their AMPK-mediated phosphorylation and their transcriptional activation of target genes, resulting in the dramatic amplification of the adiponectin-LKB1-AMPK-FOXO-signaling activation in CHIP^−/−^-livers (Fig. 8B), as indeed corroborated by our own findings (Fig. 5). Consequently, ATP-consuming biosynthetic processes would be turned off, ATP-generating catabolic processes turned on^35^, and antioxidant responses ushered that directly counteract and thus attenuate the concurrent pro-NAFLD/NASH scenario induced by hepatic CYP2E1-ROS-JNK1-activation. Thus, only around 12 months of age, when insulin resistance coupled with hepatocyte “dropout” stemming from progressive cardiac failure^15^ and consequent central venous congestion leads to the failure of these relevant hepatoprotective mechanisms, do CHIP^−/−^-livers finally succumb to NASH-like macrovesicular steatosis and cell injury.

In this scenario, FOXO transcription factors, strategically positioned relay nodes at critical intersections of these cellular signaling networks, play a key role^18^,^19^,^20^,^21^,^22^,^23^ (Fig. 8B). Accordingly, the predominant nuclear (rather than cytosolic) accumulation of pFOXO1/pFOXO3-proteins elicited by the greatly enhanced adiponectin-AMPK-signaling in CHIP^−/−^-livers (Fig. 6E), indicates that such positive FOXO-mediated transcriptional up-regulation of oxidative resistance genes and *adipoR1*/*adipoR2-*expression prevailed at the least over the first 9 months of life. Furthermore, such nuclear FOXO-activation in CHIP^−/−^-livers transcriptionally up-regulated not only the autophagic/lipophagic gene *Atg14* that regulates lipophagy, a form of cytoplasmic lipid droplet autophagy, triggering the catabolic breakdown and release of lipids for cell fuel^51^, but also the expression of lipoprotein lipase (LPL), an enzyme involved in triglyceride-breakdown into FA^23^,^52^. This synergistic FOXO-mediated transcriptional *Atg14- and lpl-*up-regulation (Fig. 5A) along with the observed AMPK-FOXO-PGC1α-mediated *acox1*-up-regulation (Fig. 5A) and pACC2-attenuation (through S79-phosphorylation; Fig. 5A,B) in CHIP^−/−^-livers would synergistically enhance both lipid-breakdown and mitochondrial FA-uptake and β-oxidation, thereby promoting the anti-steatogenic effects of activated adiponectin-AMPK-FOXO-signaling, and retarding NAFLD/NASH-progression.

Collectively, our findings in the CHIP^−/−^-livers reveal that in spite of the remarkably sustained CYP2E1-ROS-JNK1-c-Jun/AP-1-activation and the associated NAFLD/NASH-pathognomonic manifestations comparable to those seen in HF- and MCD-induced NAFLD/NASH murine models^30^,^31^,^32^,^33^,^34^, these livers remain largely resistant to NASH at the least over the initial 8–9 months of life. Furthermore, CHIP^−/−^-hepatocytes show little predisposition to MCD-WME-elicited or acetaminophen-induced liver injury, in spite of this marked hepatic CYP2E1-ROS-JNK1-activation (Fig. S5). Thus, only after this concurrent salutary activation of the adiponectin-LKB1-AMPK-FOXO-signaling axis wanes (Fig. 7D), do these CHIP^−/−^-mice begin to show characteristic NASH symptoms i.e. macrovesicular steatosis and marked hepatocellular ballooning. Our findings thus suggest that the pharmacological activation of the adiponectin-AMPK-FOXO-signaling pathway may be therapeutically beneficial in counteracting NAFLD/NASH, consistent with other proposals^24^,^25^. Accordingly, an adiponectin receptor (adipoR1/adipoR2) agonist “AdipoRon”, in clinical tests for Type 2 diabetes, is apparently effective in hepatic AMPK activation and counteraction of insulin resistance^24^,^68^. The anti-diabetic drugs metformin, phenformin and thiazolidenediones exploited in NAFLD/NASH therapy are all AMPK-activators, as are many natural products i.e. salicylates, resveratrol, epigallocatechin, capsaicin, curcumin and garlic, fueling the current pharmaceutical quest for novel, even more selective AMPK-activators^35^,^69^. Regardless of the precise pharmacological approach, the findings in our CHIP^−/−^-mouse model argue that the up-regulation of the adiponectin-AMPK-FOXO-signaling pathway may be therapeutically beneficial in the presently explosive diabetes/obesity-associated NAFLD/NASH epidemic. Although the role of CHIP in human NAFLD/NASH remains to be established, our findings may be potentially clinically relevant given not only our identification of hepatic AMPKα1 both as a CHIP-target and a NAFLD/NASH decelerator, but also the recent identification of existent human CHIP-genetic polymorphisms^61^.

## Methods

### Materials

Common cell culture media, supplements, culture plasticware, and commercial sources of protease inhibitors and DEX have been reported previously^4^. The transfection reagent, X-tremeGENE HP was obtained from Roche (Indianapolis, IN) and SYBR green master mix from Lifescience Tech. (Carlsbad, CA); INH and Extract-N-Amp for mouse tissue genotyping from Sigma (St. Louis, MO); 15-F_2t_-Isoprostane ELISA kit, TBARS assay kit, ALT- colorimetric activity assay kit, and triglyceride (TG) detection assay from Cayman (Ann Arbor, MI), Oxyblot detection materials and EMSA gel shift assay from Millipore (Billerica, MA), Pathscan Intracellular Signaling array kit from Cell Signaling Technology (Danvers, MA), BSA protein assay, Pico ECL reagents, Nuclear and Cytoplasmic extraction reagents from Thermo Fisher (Pittsburgh, PA). pcDNA3-CHIP, pcDNA3-CHIP-∆U-box, pcDNA3-CHIP-∆TPR were provided by the Patterson lab. pEBG-AMPKα1 was provided by Dr. Rob Onyenwoke (North Carolina Central University). pCW45-AMPKα2 was obtained from DNA Resource Core of Harvard Medical School (Boston, MA).

### Genotyping of CHIP-knockout mice

Male CHIP^+/−^- and female CHIP^−/−^-mice generated as described^14^,^15^ were bred and the progeny genotype verified through PCR analyses of mouse-tail genomic DNA and CHIP-protein immunoblotting (**IB**) analyses of hepatocyte lysates (Fig. S2A). Mice were fed a standard laboratory chow-diet, given water *ad libitum* and maintained under a normal diurnal light cycle. All animal experiments were carried out strictly by protocols specifically approved by the UCSF/Institutional Animal Care and Use Committee (IACUC) and its care and use of laboratory animal guidelines. CHIP^−/−^-mice were maintained from birth to ≈12 months, as their median lifespan is <1 year^14^. We employed 2–9 month-old male mice, with 1 year-olds included whenever feasible.

### Mouse hepatocyte culture and oxidative stress analyses

Cells from CHIP^+/+^-, CHIP^−/−^-, or CHIP^+/−^-livers were cultured as described^4^, with the CYP3A-inducer DEX (10 μM), or CYP2E1-inducer INH (1 mM), supplemented daily in WME for 5 days, to restore basal P450 loss upon culture. Upon harvesting, cell lysates were prepared as described^4^.

Four commonly employed oxidative stress indices^70^ were monitored: ROS-triggered membrane lipid-peroxidation 15-F_2t_-IP***-***products were assayed in the culture medium by a fluorescent immunoassay as per the manufacturer’s instructions. Levels of MDA, a byproduct of unsaturated FA-oxidation, were monitored in cell lysates as thiobarbituric acid-reactive adducts^70^. 4-HNE, another reactive unsaturated FA-oxidation byproduct, that covalently binds to protein lysine and/or SH-groups was monitored via cell lysate IB analyses^69^, and confocal immunofluorescence analyses of cultured hepatocytes *in situ* with a HNE-specific antibody^4^. Protein carbonyl-oxidation was assayed via Oxyblot (DNP-IB) analyses.

### Fluorescence-based P450 functional assays

CYP3A and CYP2E1 function was assessed by diagnostic assays of BFC 7-O-debenzylation and MFC 7-O-demethylation, respectively, as described^4^. The P450 functional contribution was assessed by preincubating cultures for an hour with KTZ (5 or 10 μM) or 4-MP (1.25 or 2.5 mM), relatively selective inhibitors of CYP3A and CYP2E1, respectively^6^, before functional assays.

### Nuclear/cytoplasmic extraction and FOXO1/FOXO3-immunoprecipitation (IP)

Nuclear and cytoplasmic subfractions were prepared from cultured hepatocytes washed with chilled phosphate-buffered saline (PBS) for 10 min with nuclear/cytosolic extraction reagents (NER/CER) (Pierce Biotech., Rockford, IL) according to the manufacturer’s instructions. Briefly, cells were mixed with ice-cold CER-I and then incubated on ice for 10 min. CER-II was then added and the cell mixture further incubated on ice for 1 min. Nuclei were harvested by centrifugation (100 × g), and the resulting supernatant was collected as the cytoplasmic extract. Nuclear pellet was suspended with ice-cold NER buffer, and nuclear protein was extracted at 4 °C for 1 h, sedimented at 16,000 × g for 10 min at 4 °C to obtain the supernatant (nuclear extract). Total protein (200 μg) from each subfraction was precleared with protein A/G-Sepharose (10 μl; Santa Cruz Biotechnology, Santa Cruz, CA) for 1 h and then immunoprecipitated with anti-FOXO1 or FOXO3 antibody (2 μg) on a rocking platform at 4 °C for 2 h, followed by the addition of protein A/G-Sepharose beads (20 μl) at 4 °C for 16 h as described^4^. Immunoprecipitated complexes were washed, eluted, and subjected to SDS-PAGE coupled with **IB** analysis against anti-phospho-serine/threonine antibody.

### qRT-PCR Analyses

Real-time PCR was performed with total RNA isolated with RNeasy mini-kit (Qiagen), treated with DNase (DNA-free kit, Ambion), and reverse-transcribed with Accupower RT-PCR kit (Bioneer) for cDNA synthesis, in Power SYBR Green PCR Master Mix (Applied Biosystems; final 25 μl-volume) with Agilent Mx3005P System. Adipose tissue *adipoQ* was similarly analyzed with total RNA extracted using a combined TRIZOL (Invitrogen, Carlsbad, CA) and RNeasy mini-kit protocol. Relative gene expression level was determined by normalizing its threshold cycle (*Ct*) to that of *Gapdh Ct*. Primers used are listed (Supplementary Information, Table S1).

### IB analyses

Upon harvesting, cultured hepatocytes were washed once with ice-cold PBS for 10 min and lysed in a cell-lysis buffer (Cell signaling Tech., Beverly, MA). The whole-cell lysates were clarified by centrifugation at 12,000 × g for 10 min. Protein concentrations were measured using the bicinchoninic acid (BCA) protein assay reagent (Pierce), 10 μg of proteins were resolved by 4–15% gradient SDS-PAGE, transferred to nitrocellulose membrane (Bio-Rad, Hercules, CA), blocked in 5% skim-milk for 1 h, and probed with primary antibodies for 16 h at 4 °C, washed with 1X Tris-buffered saline containing 0.1% Tween 20 for >5 times. Following incubation with anti-mouse IgG HRP-linked antibody or anti-rabbit IgG HRP-linked antibody, bound immunoglobulins were detected using enhanced chemiluminescence (Pierce). Antibodies used for this study and their commercial sources are provided (Supplementary Information, Table S2). Immunoblots were densitometrically quantified by ImageJ (NIH) analyses with available software (http://rsbweb.nih.gov/ij/), using corresponding actin-loading controls for normalization.

### Histological analyses

Mouse liver sections were fixed, stained, and subjected to light-microscopy and imaging by the UCSF Liver Center Pathology and Gladstone Institute, Histology and Light Microscopy Cores.

### Electromobility shift assays (EMSA)

Hepatic nuclear extracts were probed with a biotin-labeled oligonucleotide specific for the NF-κB-consensus sequence, as detailed^71^.

### Statistical analyses

Experiments were generally performed in triplicate. Data were compared by analysis of variance, and *p* values <0.05 were considered statistically significant.

## Additional Information

**How to cite this article**: Kim, S.-M. *et al.* Chip^−/−^-Mouse Liver: Adiponectin-AMPK-FOXO-Activation Overrides CYP2E1-Elicited JNK1-Activation, Delaying Onset of NASH: Therapeutic Implications. *Sci. Rep.* **6**, 29423; doi: 10.1038/srep29423 (2016).

## Supplementary Material

Supplementary Information
