# Supplementary material for: CHIP−/−-Mouse Liver: Adiponectin-AMPK-FOXO-Activation Overrides CYP2E1-Elicited JNK1-Activation, Delaying Onset of NASH: Therapeutic Implications
Source: Sci Rep. 2016 Jul 12;6:29423. doi: 10.1038/srep29423 (PMC4942616; doi:10.1038/srep29423)
Supplement: Supplementary Information [file srep29423-s1.pdf]

SUPPLEMENTARY INFORMATION

CHIP$^{-/-}$-MOUSE LIVER: ADIPONECTIN-AMPK-FOXO-ACTIVATION OVERRIDES CYP2E1-ELICITED JNK1-ACTIVATION, DELAYING ONSET OF NASH: THERAPEUTIC IMPLICATIONS

Sung-Mi Kim[1], James P. Grenert[2,6], Cam Patterson[3], and Maria Almira Correia[1,4,5,6*]

Table S1. Primers used for qRT-PCR analyses

Gene	Sequences 5'→3'
**ATP-binding cassette transporter (*abc-a1*)**	GGGAAGGACATTCGCTCG<br>TTGCTTTTCAGCTTGCTCGG
**fatty acid synthase (*fas*)**	TGCTCCCAGCTGCAGGC<br>GCCCGGTAGCTCTGGGTGTA
**insulin-induced gene 1 (*insig1*)**	TGCAGATCCAGCGGAATGT<br>CCAGGCGGAGGAGGAGATG
insig2	TGTGAGCTGGACTAGCTTGCT<br>CCTAAGCCGTAAAACAAAATG
**sterol regulatory element binding transcription factor 1c *srebp1c***	GGAGCCATGGATTGCACATT<br>GCTTCCAGAGAGGAGGCCAG
srebp2a	CGGAAGCTGTCGGGGTAG<br>GTTGTTGATGAGCTGGAGCA
**insulin receptor substrate 1 (*irs1*)**	GCGGGCTGACTCCAAGAAC<br>GCTATCCGCGGCAATGG
**stearoyl-coenzyme A desaturase (*scd1*)**	CATCATTCTCATGGTCCTGCT<br>CCCATTCGTACACGTCATTTT
**tumor necrosis factor α (*tnfα*)**	CACCACCATCAA GGACTCAA<br>AGGCAACCTGACCACTCTCC
**interleukin 1 β (*il-1β*)**	CTTTGAAGTTGACGGACCC<br>TGAGTGATACTGCCTGCCTG
**interleukin 6 (*il-6*)**	ACAACCACGGCCTTCCCTACTT<br>CACGATTTCCCAGAGAACATGT
**chemokine (C-C motif) ligand 1 (*mcp1*)**	CAGGTCCCTGTCATGCTTCT<br>GAGGATCACCAGCAGCAGGT
adipoQ	TGTTCCTCTTAATCCTGCCCA<br>CCAACCTGCACAAGTTCCCTT
**adiponectin receptor 1 (*adipoR1*)**	ACGTTGGAGAGTCATCCCGTAT<br>CTCTGTGTGGATGCGGAAGAT
adipoR2	GGAGTGTTCGTGGGCTTAGG<br>GCAGCTCCGGTGATATAGAGG
**acetyl-CoA carboxylase 1 (*acc1*)**	CCTCCGTCAGCTCAGATACA<br>TTTACTAGGTGCAAGCCAGACA
**acetyl-CoA carboxylase 2 (*acc2*)**	CCAGTCTTCCGTGCCTTTGTAC<br>CTCATCCCTCGCTCTGAACG
**acyl-CoA oxidase 1 (*acox1*)**	TCCAGACTTCCAACATGAGGA<br>CTGGGCGTAGGTGCCAATTA
**peroxisome proliferator-activated receptor γ coactivator 1α (*pgc1a*)**	CCCTGCCATTGTTAAGACC<br>TGCTGCTGTTCCTGTTTTC
**acetylcholinesterase (*ache*)**	CTATGCCTACATCTTTGAAC<br>GAGCGCTGCTCAGACCTGTG
**autophagy-related 14 (*atg14*)**	GCAGCTCGTCAACATTGTGT<br>TGCGTTCAGTTTCCTCACTG
**lipoprotein lipase (*lpl*)**	AGGGCTCTGCCTGAGTTGTA<br>AGAAATTTCGAAGGCCTGGT
**stress-responsive genetic regulator sirtuin 1 (*sirt1*)**	GCAGGTTGCGGGAATCCAA<br>GGCAAGATGCTGTTGCAAA
**glucose-regulated proteins 78 (*grp78*)**	CATGGTTCTCACTAAAATGAAAG<br>GCTGGTACAGTAACAACTG
**glyceraldehyde 3-phosphate dehydrogenase (*gapdh*)**	ACCACAGTCCATGCCATCAC<br>CACCACCCTGTTGCTGTAGCC

qRT-PCR analyses: *These were carried out as detailed (Bobard A et al. Differential regulation of sterol regulatory element-binding protein 1c transcriptional activity by insulin and liver X receptor during liver development. J Biol Chem. 2005; 280:199-206).*

Table S2. Specific antibodies used in immunoblotting (IB)-analyses

Antibody	Catalog number	Type	Dilution (v/v)	Source
CHIP	SC66830	Rabbit polyclonal	1:2000	Santa Cruz Biotech
Myc	H1314	Mouse monoclonal	1:500	Santa Cruz Biotech
HNE	MA3249	Mouse monoclonal	1:500	R&D Systems
ASK1	SC7931	Rabbit polyclonal	1:500	Santa Cruz Biotech
ASK1(pT845)	SC109911	Rabbit polyclonal	1:250	Santa Cruz Biotech
MKK4	9152S	Rabbit polyclonal	1:1000	Santa Cruz Biotech
MKK4(pS257)	4514S	Rabbit monoclonal	1:500	Cell Signaling
JNK	9252S	Rabbit polyclonal	1:2000	Cell Signaling
JNK(pT183/Y185)	4668S	Rabbit monoclonal	1:500	Cell Signaling
ATF2	SC187	Rabbit polyclonal	1:1000	Santa Cruz Biotech
ATF2 (pT71)	5112S	Rabbit monoclonal	1:500	Cell Signaling
c-Jun	SC1694	Rabbit polyclonal	1:1000	Santa Cruz Biotech
c-Jun (pS63)	2361S	Rabbit monoclonal	1:500	Cell Signaling
Akt	MAB2055	Mouse monoclonal	1:2000	R&D Systems
Akt(pT308)	MAB7419	Mouse monoclonal	1:1000	R&D Systems
Akt(pS473)	AF887	Rabbit polyclonal	1:1000	R&D Systems
LKB1	MAB8055	Mouse monoclonal	1:2000	R&D Systems
LKB1(pS428)		Mouse monoclonal	1:1000	R&D Systems
AMPK	5831S	Rabbit monoclonal	1:1000	Cell Signaling
AMPK(pT172)	2535S	Rabbit monoclonal	1:1000	Cell Signaling
ACC	3676S	Rabbit monoclonal	1:1000	Cell Signaling
ACC(pS79)	11818S	Rabbit monoclonal	1:1000	Cell Signaling
IRS1	MAB8055	Mouse monoclonal	1:2000	R&D Systems
IRS1(pS307)	44813G	Rabbit polyclonal	1:1000	Life Tech
IRS1(pY895)	3070	Rabbit polyclonal	1:1000	Santa Cruz Biotech
Phospho-S/T	9631S	Rabbit polyclonal	1:250	Cell Signaling
Sirt1	SC15404	Rabbit polyclonal	1:500	Santa Cruz Biotech
Adiponectin	2789S	Rabbit monoclonal	1:500	Cell Signaling
AdipoR2	SC46754	Goat polyclonal	1:1000	Santa Cruz Biotech
FOXO1	SC11350	Rabbit polyclonal	1:500	Santa Cruz Biotech
FOXO3	PA1805	Rabbit polyclonal	1:500	Affinity Bioreagents
NLRP3(Cryopyrin)	SC66846	Rabbit polyclonal	1:500	Santa Cruz Biotech
HA	SC-805	Rabbit polyclonal	1:400	Santa Cruz Biotech
6XHis	NBP2-31055	Mouse monoclonal	1:1000	Novus
GST	27457701	Goat polyclonal	1:1000	GE Life Science
Histone H3	AB1791	Rabbit polyclonal	1:5000	Abcam
Gapdh	SC25778	Rabbit polyclonal	1:2000	Santa Cruz
Actin	A5316	Mouse monoclonal	1:2000	Sigma

Supplementary Figure Legends:

Fig. S1. CYP2E1 stabilization in cultured rat hepatocytes upon lentiviral shRNAi-mediated CHIP-knockdown. Rat hepatocytes were cultured in the presence of EtOH (100 mM), a CYP2E1 inducer. For experimental details see Kim et al. (4).

Fig. S2. Functional stabilization of hepatic CYPs 3A and 2E1 upon genetic CHIP-ablation. **A**. Genotyping analyses of WT (+/+), hetero- (+/-) and homozygous (-/-) CHIP-mice. DNA bands corresponding to WT (950 bp) and CHIP$^{-/-}$ (550 bp) are shown (left), and IB-analyses of corresponding liver lysate CHIP (right). **B**. Age-dependent hepatic CYP3A- and CYP2E1-stabilization in CHIP$^{+/+}$- and CHIP$^{-/-}$-livers. **C**. Functional CYP3A- and CYP2E1-stabilization assessed *in situ* in cultured hepatocytes from 2-month-old mice with diagnostic probes.

Fig. S3. PathScan intracellular-signaling array analyses of hepatocytes from CHIP$^{+/+}$- and CHIP$^{-/-}$-mice. (**A**). Target map of the Pathscan arrays. 1-Positive control; 2-Negative Control; 3-ERK1/2 Thr202/Tyr204-Phosphorylation; 4-Stat1 Tyr701-Phosphorylation; 5-Stat3 Tyr705-Phosphorylation; 6-Akt Thr308-Phosphorylation; 7-Akt Ser473-Phosphorylation; 8-AMPKα-Thr172-Phosphorylation; 9-S6 Ribosomal Protein Ser235/236-Phosphorylation; 10-mTOR Ser2448-Phosphorylation; 11-HSP27 Ser78-Phosphorylation; 12-Bad Ser112-Phosphorylation; 13-p70 S6 Kinase Thr389-Phosphorylation; 14-PRAS40 Thr246-Phosphorylation; 15-p53 Ser15-Phosphorylation; 16-p38 Thr180/Tyr182-Phosphorylation; 17-SAPK/JNK Thr183/Tyr185-Phosphorylation; 18-PARP Asp214-Cleavage; 19-Caspase-3 Asp175-Cleavage; 20-GSK-3β-Ser9-Phosphorylation. Representative Pathscan array analyses of hepatocyte lysates from 2-, 4- and 9-month-old CHIP$^{+/+}$- and CHIP$^{-/-}$-mice. Images were acquired upon brief exposure of the slide to chemiluminiscent film. (**B**). Densitometric quantification (Mean ± SD) of PathScan films from hepatocyte lysates from 9-month-old mice, normalized against the positive control (+CT) (N = 3 individual liver lysates). Statistically significant differences between values shown at p <0.005 (§).

Fig. S4. Relative antilipogenic, prolipogenic and proinflammatory gene expression in $CHIP^{+/+}$- and $CHIP^{-/-}$-livers. qRT-PCR analyses of total RNA extracted from intact $CHIP^{+/+}$- and $CHIP^{-/-}$-livers at 2- or 9-months. Relative mRNA expression of antilipogenic (*insig-1, insig-2*), prolipogenic (*srebp-1c, srebp-2a, abc-a1, fas, scd-1, acc1*) and proinflammatory/inflammatory cytokines (*tnf-α, il-6, il-1β*) and chemokine (*mcp-1*) genes was determined as described (Experimental Procedures). Statistical significance between the values shown at $p<0.001$ (*) or $p< 0.005$ (§).

Fig. S5. Predisposition of $CHIP^{+/+}$- and $CHIP^{-/-}$-livers to cell-injury upon toxigenic insults. Relative time-dependent cell injury as monitored through extracellular leakage of cytosolic ALT into the medium of INH-pretreated hepatocytes cultured in regular WME (CT) or MCD-WME (MCD) on the 4th day of culture, or treated with acetaminophen at a hepatotoxic 5 mM-concentration[44]. Statistical significance between the values shown at $p<0.001$ (*) or $p< 0.005$ (§).

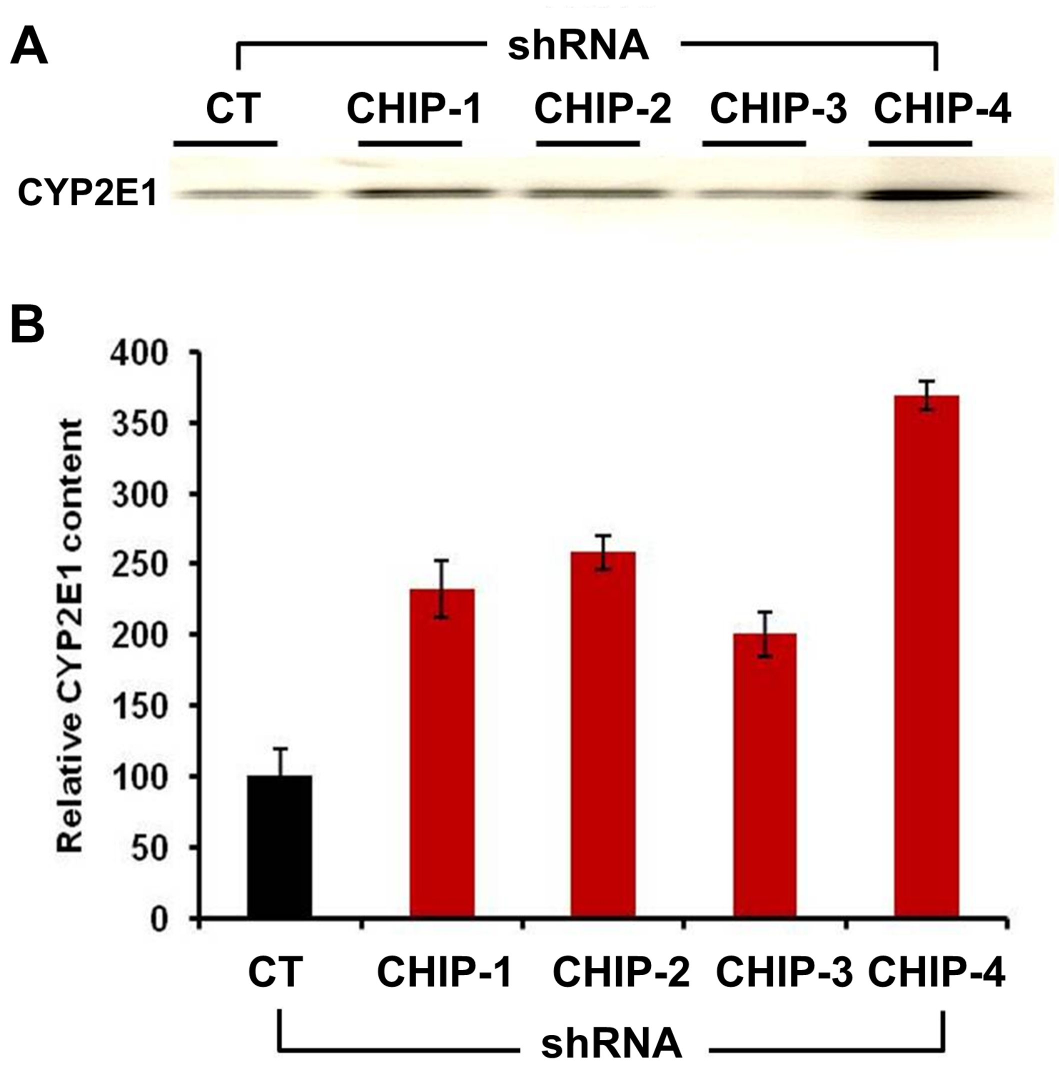

Figure-S1 (Correia)

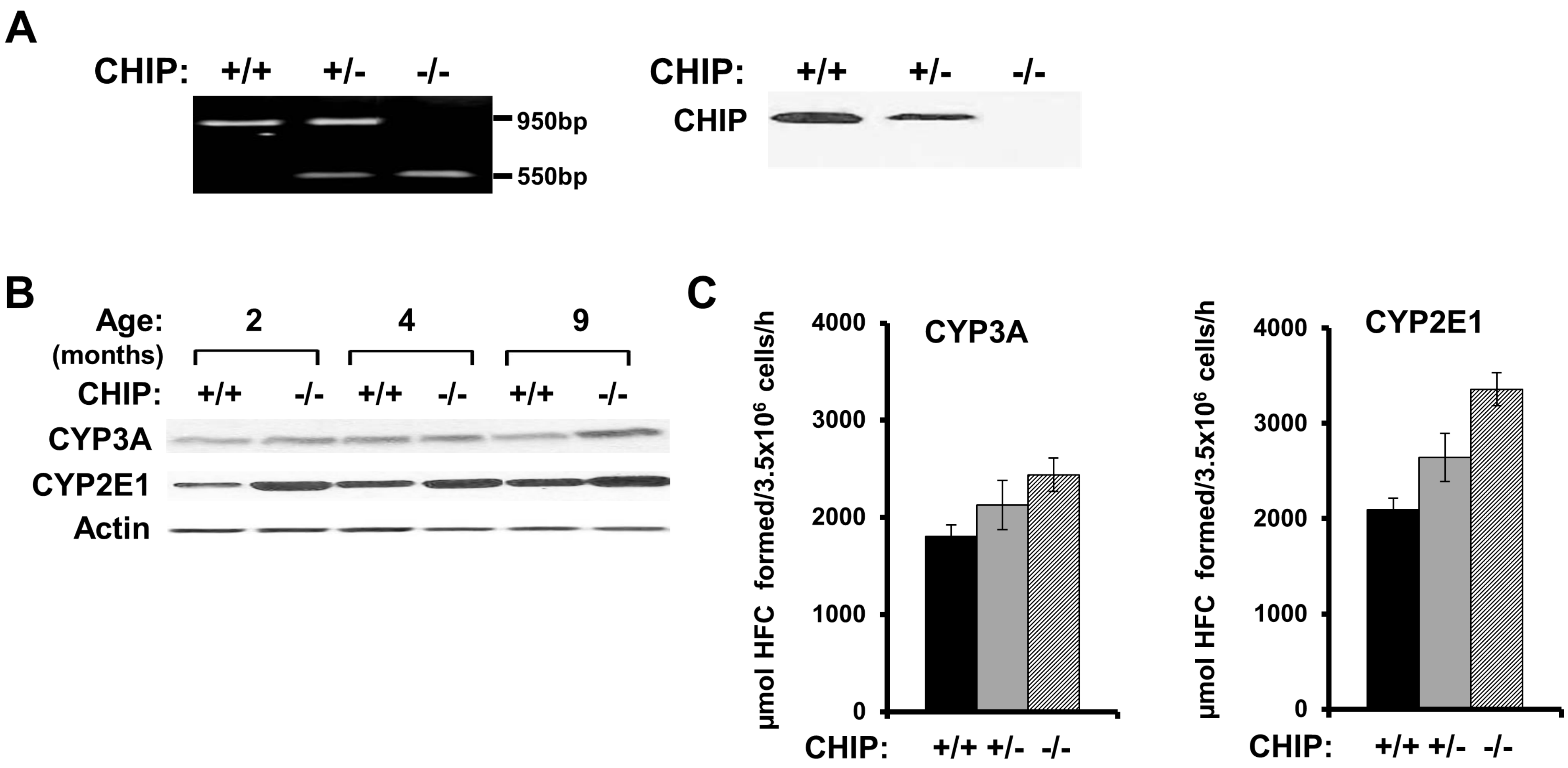

Figure-S2 (Correia)

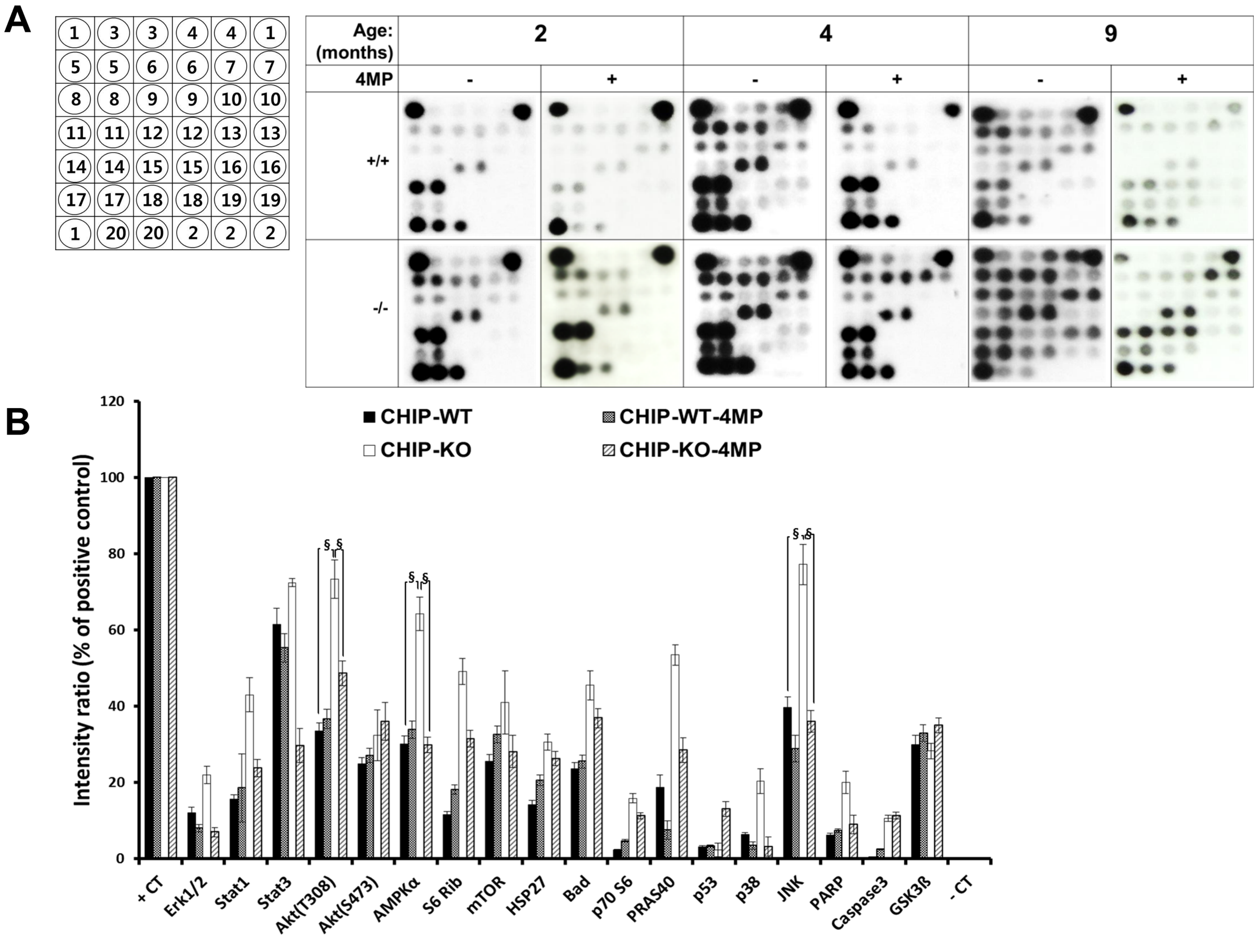

Figure-S3 (Correia)

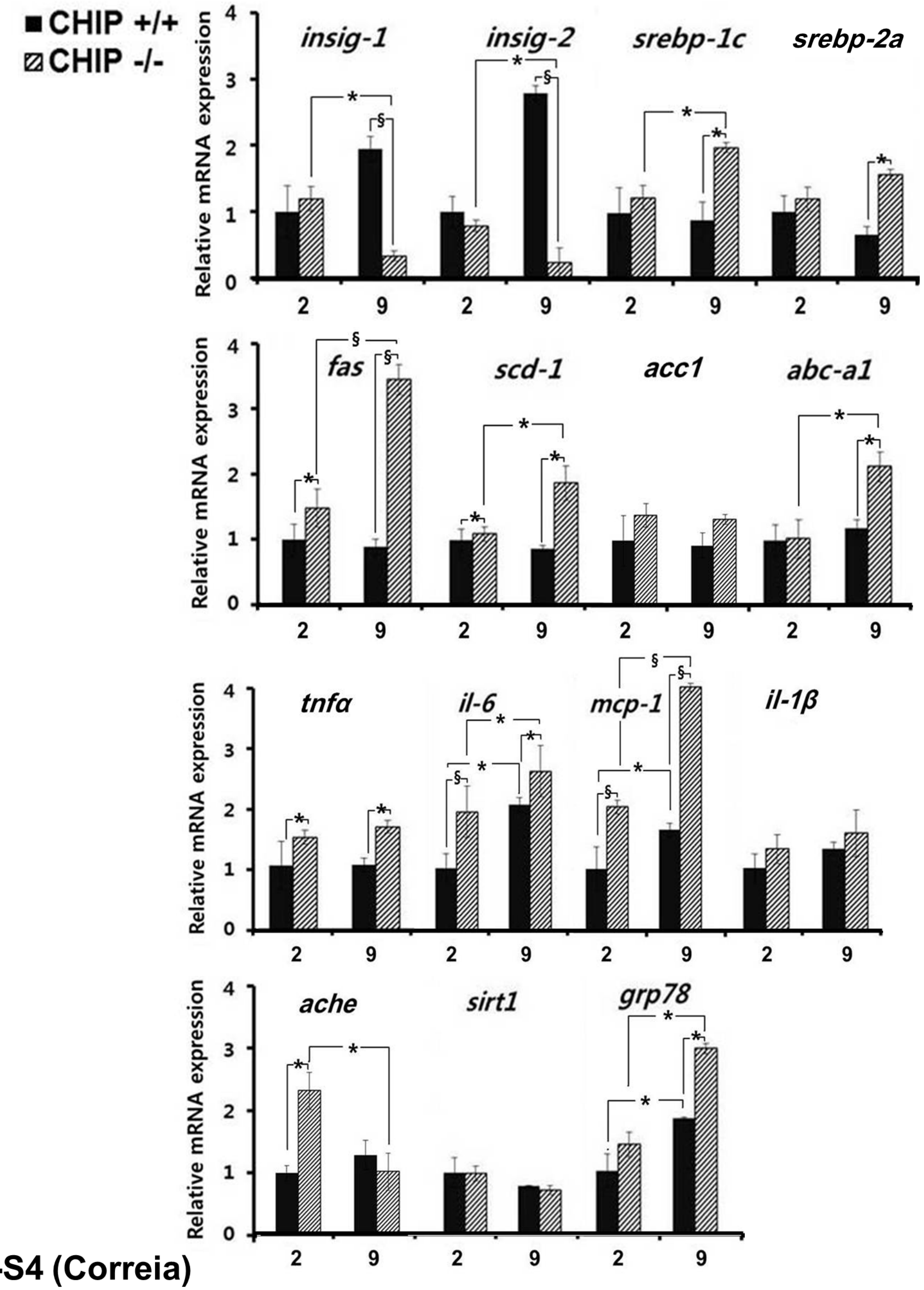

Figure-S4 (Correia)

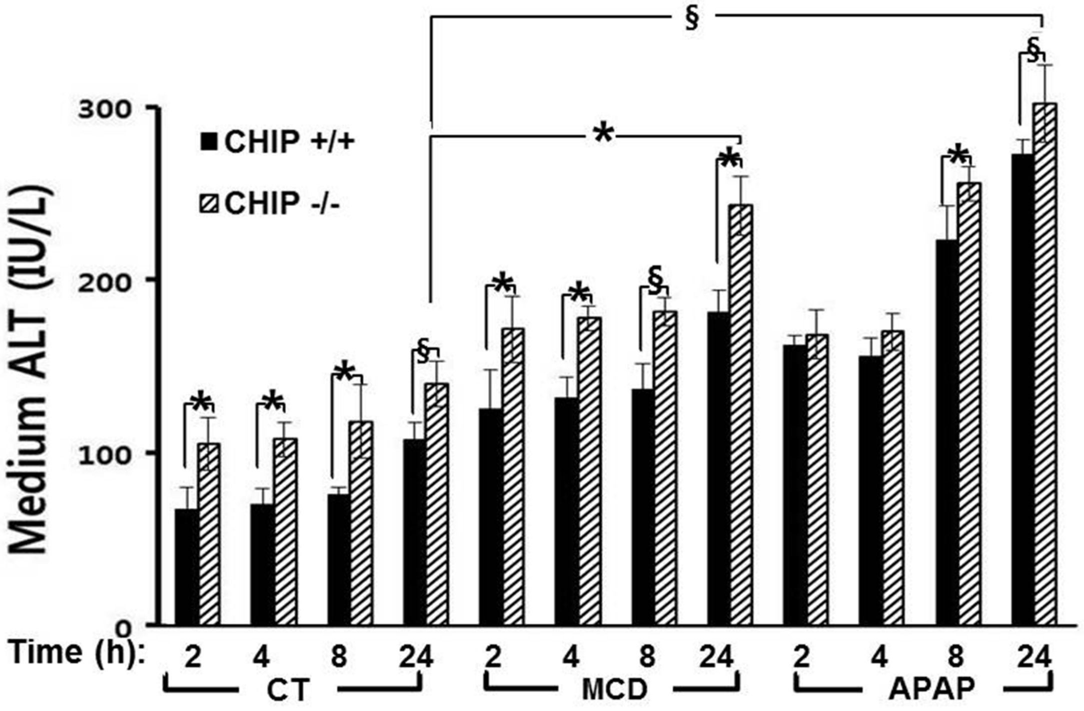

Figure-S5 (Correia)
